# CFTR modulators response of S737F and T465N CFTR variants on patient-derived rectal organoids

**DOI:** 10.1186/s13023-024-03334-3

**Published:** 2024-09-13

**Authors:** Karina Kleinfelder, Paola Melotti, Anca Manuela Hristodor, Cristina Fevola, Giovanni Taccetti, Vito Terlizzi, Claudio Sorio

**Affiliations:** 1 Department of Medicine, Division of General Pathology, Cystic Fibrosis Laboratory “D. Lissandrini”, University of Verona, 37134 Verona, Italy; 2 Cystic Fibrosis Centre, Azienda Ospedaliera Universitaria Integrata Verona, 37126 Verona, Italy; 3 Department of Pediatric Medicine, Meyer Children’s Hospital IRCCS, Cystic Fibrosis Regional Reference Center, Florence, Italy

**Keywords:** Human intestinal monolayers, CFTR modulators, Electrophysiological measurements, Rare mutations, Personalized medicine

## Abstract

**Background:**

Predictions based on patient-derived materials of CFTR modulators efficacy have been performed lately in patient-derived cells, extending FDA-approved drugs for CF patients harboring rare variants. Here we developed intestinal organoids from subjects carrying S737F- and T465N-CFTR in trans with null alleles to evaluate their functional impact on CFTR protein function and their restoration upon CFTR modulator treatment. The characterization of S737F-CFTR was performed in two subjects recently assessed in nasal epithelial cells but not in colonoids.

**Results:**

Our functional analysis (Ussing chamber) confirmed that S737F-CFTR is a mild variant with residual function as investigated in colonoids of patients with S737F/Dele22-24 and S737F/W1282X genotypes. An increase of current upon Elexacaftor/Tezacaftor/Ivacaftor (ETI) treatment was recorded for the former genotype. T465N is a poorly characterized missense variant that strongly impacts CFTR function, as almost no CFTR-mediated anion secretion was registered for T465N/Q39X colonoids. ETI treatment substantially improved CFTR-mediated anion secretion and increased the rescue of mature CFTR expression compared to either untreated colonoids or to dual CFTR modulator therapies.

**Conclusions:**

Our study confirms the presence of a residual function of the S737F variant and its limited response to CFTR modulators while predicting for the first time the potential clinical benefit of Trikafta® for patients carrying the rare T465N variant.

## Introduction

Cystic fibrosis transmembrane conductance regulator (CFTR) is ATP-gated anion channel involved in the regulation of salt and water transport across a variety of epithelium [1, 2], presenting a major role in the maintenance of whole-body fluid homeostasis [3]. The impaired function of the CFTR channel causes cellular alkalinity and luminal acidification and generates the accumulation of viscous mucus, mainly in the lungs and gastrointestinal tract, which leads to organ dysfunction and cystic fibrosis (CF) disease manifestation [4, 5]. CF is the most common monogenetic disease in the Caucasian population, caused by variants on the CFTR gene that may lead to reduction or abolishment of anion secretion. A condition that is dependent on the defect mechanisms of the CFTR protein (defective synthesis/expression, folding flaws, impaired gating or conductance of the channel, reduced quantity and peripheral stability defects) [6]. The variety of severity of CFTR defects at the molecular level correlates with a range of clinical manifestations. In general, variants that incite absent or very weak residual CFTR activity provoke more severe CF disease, whereas variants that lead to relatively high residual function are associated with milder CF phenotype [7, 8]. Currently, among the 2100 different variants identified in the CFTR gene, 719 of them have confirmed the CF disease liability based on the CFTR2 Database (https://cftr2.org/, accessed on January 09th, 2024). Many of these variants can attribute multiple defects to the CFTR channel, requiring the combined use of CFTR-targeted therapies that, in the last decade, have significantly advanced the pathophysiological condition of CF patients [9].

These highly effective mutation-based pharmacotherapies are capable of correcting the basic defect of the CFTR protein but are still not eligible for all CF patients, principally for those that harbor rare or ultra-rare CFTR variants (10–15% of patients) whose responsiveness to modulators is still unknown [10]. Because of the very low patient numbers, the classical clinical trials have been replaced by using models based on patients-derived materials to predict responses to CFTR modulator drugs at an individual level [11, 12]. Moreover, the US Food and Drug Administration (FDA) and the European Medical Authorities (EMA) decided to approve the label extension of existing CFTR modulators based on data generated with the use of in vitro data [13, 14]. The use of primary patient-derived cell cultures such as human intestinal organoids [15] and human nasal epithelial cells [16] are feasible and robust models and have been demonstrated to be more reliable predictive tools than heterologous expression systems [17, 18]. Intestinal organoids culture presents a potential application in the CF field and has been used for supporting diagnosis with CFTR-function measurements, and for theratyping applications [19–22]. As a predictive in vivo drug response platform, human intestinal organoids have been used in both formats: 3D, to measure CFTR-mediated fluid influx into the lumen of rectal organoids [23, 24] and, in a so-called 2D, where rectal organoids are dissociated into single cells and grown in a Transwell® device, suitable for the measurement of transepithelial currents [25, 26]. Recently, Birimberg-Schwartz and colleagues [27], through a direct comparison of electrophysiological measurement of the intestine and nasal cells, further confirmed the suitability of 2D intestinal monolayers for theratyping applications in CF showing a larger measurable CFTR functional range in response to agonists in comparison to nasal cells.

In this study, we report the data from our functional studies on S737F- and T465N-CFTR variants performed on patient-derived intestinal organoids. The S737F (c.2210C > T; p.Ser737Phe) is a CFTR missense variant, located in exon 13 and characterized by a substitution of serine with phenylalanine in position 737, eligible for Trikafta or Symdeco treatment in USA according to the FDA approval. S737F is associated with hypochloremic alkalosis and mild CF phenotype [28] with conflicting interpretations of pathogenicity. The functional impact of S737F and responsiveness to modulators in the same individuals was recently characterized in nasal epithelial cells (hNEC) [29] but not in colonoids. On the other hand, the T465N (c.1394C > A, p.Thr465Asn) is a pathogenic variant poorly characterized, not yet present in the CFTR2 database (https://cftr2.org/, accessed on January 09th, 2024). This missense variant results in a substitution of threonine to asparagine in position 465, which could affect the NBD1 domain of the CFTR protein. It is located in exon 11 and may present class II and III defects with no FDA-authorized treatment [30]. This variant was found in Asian, Hispanic, and Caucasian populations, and it is associated with severe CF phenotype, pancreatic insufficiency, and congenital absence of the vas deferens (CAVD) [31–33]. Preliminary data on CFTR function following treatment with CFTR modulators indicate a borderline response to Elexacaftor/Tezacaftor/Ivacaftor (ETI) Trikafta® treatment (10.1101/2023.07.07.548159). Hence, here we aimed at: (a) the theratyping T465N variant in response to CFTR modulators in human colonoids and (b) evaluating the electrophysiological current of S737F in the presence and absence of ETI Trikafta® treatment in human colonoids. The presence of class I variants (nonsense and/or deletion) on the other alleles permits the isolation of the contribution of the missense variants.

The aim of this study is to evaluate, in intestinal organoids, the response to CFTR modulators approved in clinic in order to measure the residual activity of the channel and the potential response to the treatment.

## Methods

### Biological specimen collection

Rectal biopsies were collected from CF participant with S737F/W1282X (n = 1), S737F/Dele22-24 (n = 1), T465N/Q39X (n = 1) genotypes and from five non-CF subjects that were used to provide reference for Isc values and rectal organoids morphology. Samples were taken during investigative or surveillance colonoscopy. Written informed consent was obtained from all participants according to the local ethical committee’s rules (center of Florence, ethics Committee number 194/2019 and 110/2021) (CF center of Verona CRCFC-CFTR050).

### Clinical data of patients under study

Patients compound-heterozygous for the S737F and T465N missense variants were followed in the Cystic Fibrosis Centre of Tuscany region (Italy). The diagnostic tests were performed as reported by Terlizzi and colleagues 2018 [28]. Clinical data recorded for the patients enrolled are summarized in Table 1. All patients had given consent to the recording of their clinical data and for their anonymous use for scientific purposes.

**Table 1:** Clinical characteristics of CF patients enrolled in this study

Subject	IRT ^ (ng/mL)	Age at first evaluation (m/y)	Reason for sweat testing	*CFTR* genotype	First SC value	Diagnosis/label at first evaluation	Age at 30 September 2023 (years)	Microbiology	Last BMI	Last FEV_1_	Pancreatic function	Last SC value	Diagnosis/label at study end
1	37	10 m	Hypochloremic alkalosis	S737F 22,23,24 del	71	Inconclusive diagnosis	21.5	MSSA	22.2	91	PS	89	Inconclusive diagnosis
2	64	1 m	Positive NBS	S737F W1282X	51	CRMS/CFSPID	21.8	MSSA	19.5	109	PS	121	CF
3	204	1 m	Positive NBS	T465N Q39X	109	CF	10.5	MSSA	18.5	121	PI	117	CF

CFTR: Cystic fibrosis transmembrane conductance regulator; CRMS: CFTR-related metabolic syndrome; CFSPID: CF screen positive, inconclusive diagnosis; CF: Cystic Fibrosis; SC: sweat chloride; IRT: immunoreactive trypsinogen; NBS: newborn bloodspot screening; BMI: body mass index; FEV_1_: forced expiratory volume in the 1st second; MSSA: methicillin-susceptible Staphylococcus aureus; PS: pancreatic sufficiency; PI: pancreatic insufficiency

### Crypts isolation and rectal organoids cultures

For biopsy collection, CF patients were first treated with a (sodium phosphate) enema to ensure appropriate bowl preparation and thus having a clean colon/rectum. The endoscope was introduced rectally, and four forceps biopsies were obtained on sight with a flexible endoscope (approximately 5 cm from the anal verge) for each participant to guarantee a good amount of stem cell isolation. Briefly, human rectal biopsies recovered were immediately stored at 4 °C in surgical medium (RPMI-1640, glutamax 1X, HEPES 10 mM, P/S 1%, gentamicin 10 µg/mL and ciprofloxacin 20 µg/mL). Biopsies were then washed with cold PBS solution and incubated in 10 mM EDTA for 90–120 min at 4 °C. After washing, the isolated crypts were mixed with 50% Matrigel (Corning, Corning, NY, USA) and sown in 30 µL per well (with about 20–30 crypts/10 μL matrigel/droplet) in pre-warmed 24-well plates. After matrigel polymerization for 15–30 min at 37 °C, the plated crypts were covered with pre-warmed complete medium, consisting of 15% Advanced DMEM/F12 (supplemented with 1% penicillin and streptomycin, 0.2% primocin, 10 mM HEPES and 1% Glutamax), 1 × N2, 1 × B27 (all from Invitrogen), 1.25 mM N-acetylcysteine (Sigma, Kawasaki, Japan) and the following growth factors: 50 ng mL^−1^ mouse epidermal growth factor (mEGF; invitrogen), 50% Wnt3a-conditioned medium (WCM, Laguna Beach, CA, USA) and 10% noggin-conditioned medium (NCM), 20% Rspo1-conditioned medium, 10 mM nicotinamide (Sigma, Tokyo, Japan), 10 nM gastrin (Sigma), 500 nM A83-01 (Tocris, Bristol, UK), 1 μM SB 431542 (Tocris), 10 nM PGE (Sigma) and 3 μM SB202190 (Sigma). Additional antibiotics (Gentamycin and Vancomycin, Sigma, 1:1000 50 µg/mL) were used during the first week of culture. Medium was refreshed every other day and outgrowing crypts/organoids were expanded 1:3–1:5 times every 7–10 days. Complete medium was supplemented with 10 µM Rho inhibitor (Y27623) and 10 µM Chir (GSK3 inhibitor, CHIR-99021) (both from Sigma) during the first 2 days after seeding the crypts and after passaging.

### Culture of epithelial monolayers of rectal organoids (2D)

For the culture of epithelial monolayers, matrigel-embedded human rectal organoids were suspended in advanced DMEM/F12 (4 °C; Gibco) and washed by centrifugation (5 min, 200 × g) to remove the Matrigel matrix. Intestinal organoids were dissociated by brief (75 s, 37 °C) incubation in trypsin (0.25%) solution (Gibco), followed by repeated (30x) aspiration through a 200 µL pipette tip. Dissociation was monitored by visual inspection (Olympus CKK31 inverted microscope), and the above procedure was repeated until most organoids had dissociated into small cell clusters. Trypsin activity was quenched by addition of fetal calf serum (10%) in advanced DMEM, and cells were washed in advanced DMEM and filtered through a cell strainer (70 µm; Falcon). Cells were counted using a hemocytometer and seeded (2.5 × 10^5^ cells/cm^2^) on permeable inserts (Transwell #3470; Corning) that had been pretreated with human placenta collagen, type IV (10 µg/cm^2^) (Sigma-Aldrich) diluted in saline phosphate buffer and incubated at 37 °C for at least 2 h. Culture medium was the same used for extracellular matrix-embedded organoids, except that CHIR99021 (10 μM; Sigma-Aldrich) and Y-27632 (10 μM; Sigma-Aldrich) were added during the first two days after seeding. Cells were cultured until a confluent monolayer was obtained (6–9 days). For assessing the formation of a continuous epithelial monolayer, cultures were examined by microscopy and the transepithelial electrical resistance (TEER) was monitored using chopstick electrodes (EVOM2; World Precision Instruments, Sarasota, FL, USA).

### Trans epithelial electrical resistance (TEER)

TEER was measured using an EVOM2 epithelial voltohmmeter (World Precision Instruments) before refreshing the medium. The readings of the voltohmmeter can be multiplied by the surface area of the Transwell inserts (0.33 cm^2^) to calculate the unit area of resistance (Ω cm^2^). A TEER value of 400 Ω cm^2^ was considered an index of complete monolayer formation.

### Measurements of CFTR-mediated anion secretory currents across colonoids monolayers

Electrophysiological measurements were performed directly on the filter using specific Ussing chambers (P2300) and sliders (P2302T) (Physiologic Instruments, San Diego, CA, USA) and a voltage clamp EVC4000 (World Precision Instruments, Sarasota, FL, USA). For transepithelial current measurement, the colonocyte monolayers were incubated with Elexacaftor, ELEXA (VX-445, 3 μM; Med Chem Express, Monmouth Junction, NJ, USA), Lumacaftor, LUMA (VX-809; 3 μM; Selleck Chemicals LLC, Houston, TX, USA) and Tezacaftor, TEZA (VX-661, 3 μM; Selleck Chemicals LLC, Houston, TX, USA), or vehicle (dimethyl sulfoxide, DMSO, 0.1%) for 20–24 h. Monolayers were bathed in symmetrical Meyler saline solution (pH 7.4) [10 mM Hepes; 0.3 mM Na_2_HPO_4_; 0.4 mM NaH_2_PO_4_; 1.0 mM MgCl_2_; 1.3 mM CaCl_2_; 4.7 mM KCl; 128 mM NaCl; 20.2 mM NaHCO_3_; 10 mM D-glucose] for the measurement of chloride secretion mediated by CFTR. Solutions were maintained at 37 °C, gassed with 95% O_2_, 5% CO_2_. The transepithelial potential difference was clamped at 0 mV with a VVC-MC8 module (Physiologic Instruments), and the resulting short-circuit current (Isc) was recorded using a PowerLab 8/35 AD-converter (AD Instruments, Bella Vista, Australia) and associated software (LabChart v8; AD Instruments, Bella Vista, Australia). First, short circuit current reduction was blocked by 100 µM Amiloride (M) stimulus that inhibits the sodium channel EnaC; then filters were tested with components that act positively on CFTR activity: 10 µM forskolin (Sigma) applied to both apical (ap) and basolateral (bl) surfaces and 0.3 µM VX-770 (Selleckchem) (ap + bl). The experiment was concluded with the addition of the CFTR inhibitor, 20µM PPQ-102 (Tocris), from the apical and basolateral sides. At the end, 20 µM ATP (ap + bl) were just used to assess filters viability.

### Forskolin-induced swelling (FIS assay)

Rectal organoids from a 7-day-old culture were seeded in a 96 well plate in 5 μL of 50% Matrigel (Corning) containing 30–40 organoids in 100 μL of culture medium with or without CFTR modulators: 3 µM VX-661 (Selleck Chemicals LLC, Houston, TX, USA), 3 µM VX-809 (Selleck Chemicals LLC, Houston, TX, USA) and 3 µM VX-445 (Med Chem Express) or combinations thereof. One day after seeding, organoid images were acquired at 37 °C and 5% CO_2_ humidified atmosphere in bright field at 4 × magnification every 30 min, for a total acquisition of 120 min by using EVOS Cell Imaging System (Thermo Fisher Scientific). For CFTR potentiation, 3 μM of VX-770 (Selleck Chemicals LLC) was added simultaneously with forskolin. The organoid swelling was measured (xy plane) and related to t = 0 (prior the addition of forskolin) and to forskolin treatment (Fsk 0.8 μM) as follows: starting from the image acquired at time t = 0, organoids were numbered progressively, using PowerPoint software, until selected organoids were numbered, creating a mask. The subsequent overlapping of the mask to images ensured the evaluation of the same set of organoids over time. Then, the circumference of each numbered organoid was measured using ImageJ software [34] and a freehand selections tool, and the corresponding area was automatically calculated in pixels by the software. Normalized data are expressed as total area under the curve (AUC, t = 60 min; baseline, 100%) calculated using GraphPad Prism version 7 (GraphPad Software, San Diego, California, USA). A Ordinary one-way ANOVA was used to calculate statistical differences and p < 0.05 was considered statistically significant.

### Western blot

3D organoids were lysed in RIPA/EDTA/DTT/vanadate lysis buffer (50 mM Tris pH 7.5, 150 mM NaCl, 1% Triton X-100, 1% sodium deoxycholate, 0.1% SDS, 1 mM EDTA, 1 mM DTT, 1 mM sodium orthovanadate) with protease inhibitor cocktail (Roche, Mannheim, Germany) for 30 min in ice. Soluble fractions were analyzed by SDS-PAGE on 7.5% homemade Tris–Glycine gels. After electrophoresis, proteins were wet transferred from gel to a polyvinylidene difluoride (PVDF) membrane by electrophoresis with a transfer System (Bio-Rad, Hercules, CA, USA) at constant 100 V for 60 min. The membrane was blocked with 5% non-fat dry milk protein reconstituted in Tris-buffered saline–Tween (0.3% Tween, 10 mM Tris (pH 8) and 150 mM sodium chloride in water) and probed overnight at 4 °C with human CFTR-specific antibodies (cystic fibrosis folding consortium 450, 570 and 596, 1 in 1000 dilution in blocking solution). After the washing step, the membrane was probed with goat mouse-specific horseradish peroxidase (HRP)-conjugated secondary antibodies (Cell signaling, 1:12,000 dilution in blocking solution). As a loading control, we used β-Actin detected by α-beta-Actin antibody (1:1000) (Cell signaling, 4970). The blots were developed with ECL Westar Supernova ECL substrate (Cyanagen, Bologna, Italy). The imaging was performed using ImageQuantTM LAS 4000 software (GE Healthcare, Chicago, IL, USA) in a linear range of exposure. CFTR proteins level were quantified by densitometry of immunoblots using ImageJ version 1.53t 24 August 2022.

### Patient-derived rectal 3D organoids morphology

The morphology of 3D organoids was analyzed with optic microscopy JulisTM Br (Cell History Recorder, NanoEntek, Pleasanton, USA), with 4X objective during standard culture conditions.

### Statistical analysis

Data are represented as mean ± S.D. GraphPad Prism 9.0 software (San Diego, CA, USA) was used for all statistical tests. We applied a statistical analysis based on traditional multiple comparisons to the data since each patient is considered in his or her biological specificity and uniqueness rather than as the statistical realization of a random variable. A non-parametric test, such as the Kruskal–Wallis test, was used for comparison analysis between DMSO and treatment with CFTR modulators, and p-values ≤ 0.05 were considered statistically significant.

## Results

### Evaluation of S737F-CFTR activity in organoid-derived rectal monolayers

**Fig. 1:**
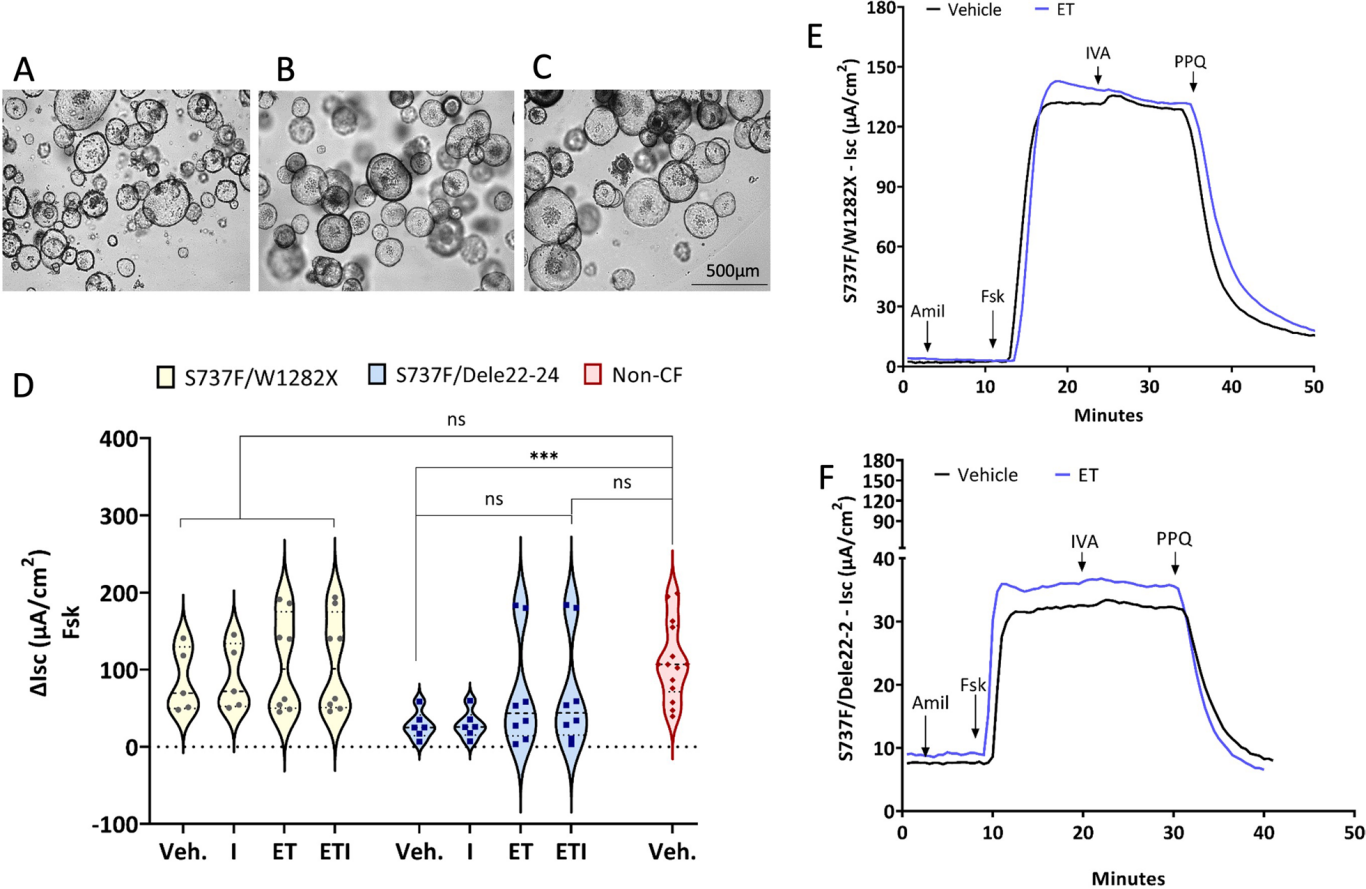
Functional evaluation of modulator treatment on rectal organoids derived from subjects 1 and 2 carrying the S737F/W1282X and S737F/Dele22-24 genotype, respectively. **A** Light microscopy analysis of the morphology of Non-CF (used here as reference), **B** S737F/W1282X and **C** S737F/Dele22-24 intestinal organoids in culture at steady-state condition. **D** Violin plot (with all points) showing the summary of results. Data reported are the amplitude of the current after addition of forskolin in colonoids treated with vehicle (Veh., DMSO), IVA (I), ELEXA/TEZA (ET) or ELEXA/TEZA/IVA (ETI). Kruskal–Wallis test, ***p = 0.0005. Modulators-treated organoids (I, ET and ETI) versus vehicle and versus non-CF group used here as reference (each diamond symbol corresponds to single ΔIsc values obtained from five non-CF subjects). Representative Isc traces of the effect of vehicle alone (DMSO; black trace), or the correctors elexacaftor + tezacaftor (ELEXA/TEZA, ET; 3 µM each; purple trace) on **E** S737F/W1282X and **F** S737F/Dele22-24 rectal organoids (derived from subject 1 and 2) with the short-circuit current technique. During the recordings, the colonoids were sequentially treated (as indicated by downward arrows) with amiloride (10 µM), forskolin (10 µM), ivacaftor (0.3 µM), and the CFTR inhibitor PPQ-102 (20 µM)

The theratyping of S737F/W1282X and S737F/Dele22-24 in response to ELEXA/TEZA/IVA combination done in patient-derived nasal cells has demonstrated a significant rescue of CFTR activity for the S737F variant [29]. The electrophysiological measurement done on matched rectal organoids grown as monolayers showed that S737F/W1282X and S737F/Dele22-24 present good residual function (DMSO-ΔIsc: 86 ± 42 µA/cm^2^ and 28 ± 18 µA/cm^2^, respectively), with mean versus mean values corresponding to approximately 77 and 25% of non-CF colonoids, respectively. The S737F/Dele22-24 variant displayed a significant difference in mean Isc compared to non-CF organoids in resting conditions. ELEXA/TEZA/IVA (ETI) treatment increased the CFTR-mediated anion secretion to Isc values that became indistinguishable from non-CF samples mostly to S737F/Dele22-24 genotype (Isc values means moved to 98% and 62% of non-CF mean for S737F/W1282X and S737F/Dele22-24, respectively). Moreover, the evaluation of the morphology of the S737F/W1282X and S737F/Dele22-24 3D organoids revealed the presence of large fluid-filled lumens similar to healthy control. Of note, CF organoids with severe mutations lack a visible lumen. The presence of luminal salt and fluid transport indicates the presence of a functional CFTR channel at the physiological concentration of cAMP [4, 19, 20] (Fig. 1). In line with these findings, the patients present a mild phenotype, as reported in Table 1, in the absence of bronchiectasis at the computer tomography scan and no congenital bilateral absence of vas deferens. Based on that, no further characterization was considered necessary for these organoids.

### T465N-CFTR characterization in response to CFTR modulators using patient-derived rectal organoids

**Fig. 2:**
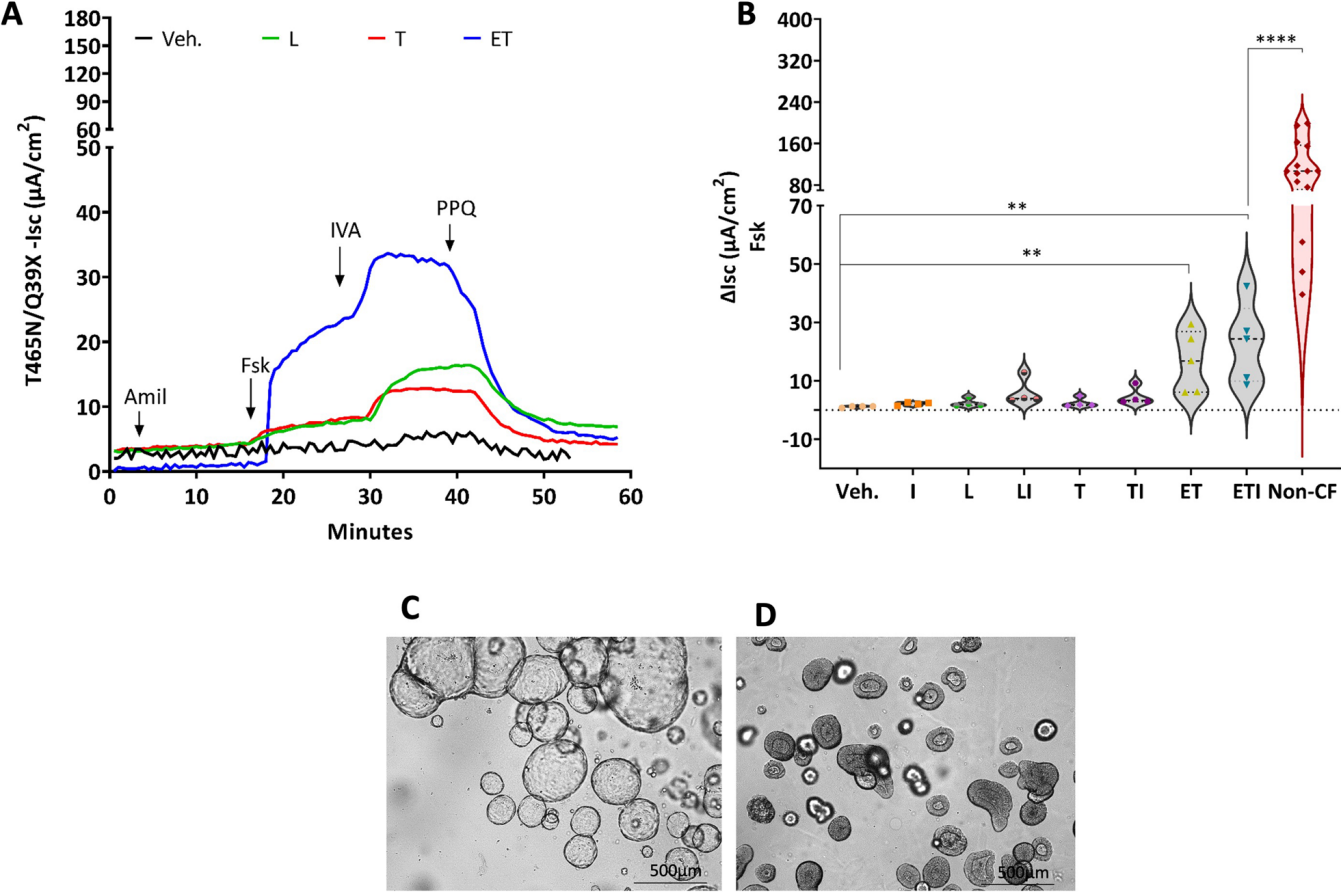
Functional evaluation of modulator treatment on T465N/Q39X rectal organoids. **A** Representative Isc traces of the effect of the vehicle alone (DMSO; black trace), or the correctors LUMA (L, green trace), TEZA (T, red trace) and ELEXA/TEXA; (ET, 3 µM each; blue trace). During the recordings, the colonoids were sequentially treated (as indicated by downward arrows) with amiloride (10 µM), forskolin (10 µM), ivacaftor (0.3 µM), and the CFTR inhibitor PPQ-102 (20 µM). **B** Violin plot (with all points) showing the summary of results. Data reported are the maximal amplitude of the current after addition of forskolin in colonoids pre-treated with vehicle (Veh.) or CFTR modulators as indicated: IVA (I), LUMA (L), LUMA/IVA (LI), TEZA (T), TEZA/IVA (TI), ELEXA/TEZA (ET) and ELEXA/TEZA/IVA (ETI). Non-CF mean of ΔIsc values from a minimum of three independent experiments obtained from five non-CF subjects (red violin plot) are shown here as reference. Kruskal–Wallis test, **p ≤ 0.007, modulators-treated organoids versus vehicle (DMSO) and **** p < 0.0001, ELEXA/TEZA/IVA-treated organoids versus Non-CF organoids. **C** Light microscopy analysis of the morphology of Non-CF (used here as reference) and (**D**) T465N/Q39X intestinal organoids in culture at steady-state condition

We then investigated the response to CFTR modulators of the rare and uncharacterized variant T465N using rectal organoids derived from a CF patient carrying the T465N/Q39X genotype. This patient was diagnosed based on a positive neonatal screening and presented with a CF phenotype with pancreatic insufficiency (Table 1). The CFTR-mediated transepithelial anion secretion in untreated intestinal monolayers displayed an almost null CFTR activity (1 ± 0.3 µA/cm^2^) elicited by forskolin. 24 h pre-treatment with correctors such as Lumacaftor (L) and Tezacaftor (T) increased the CFTR-mediated anion secretion: L/I-ΔIsc 6 ± 5 µA/cm^2^ and T/I-ΔIsc 5 ± 3 µA/cm^2^ respectively. However, a strong increment of CFTR function was registered only with the combined E/T treatment reaching, with the acute addition of I, the Isc sum of 23 ± 14 µA/cm^2^ (Fig. 2A, B), a value that corresponds to about 20% of the non-CF reference samples (mean vs mean). The morphology of untreated T465N/Q39X 3D organoids confirmed the CF phenotype with barely detectable lumen (Fig. 2C, D).

**Fig. 3:**
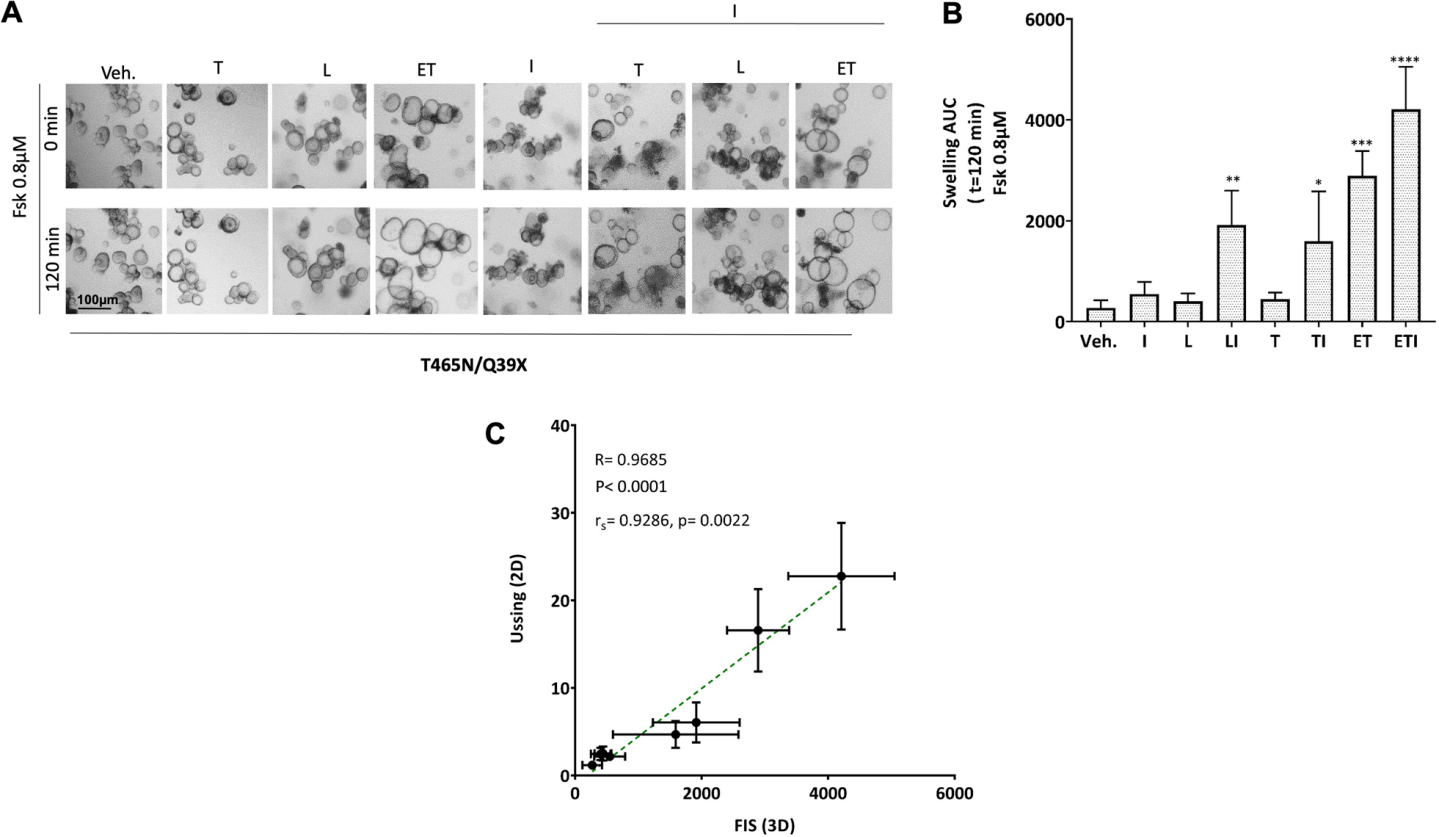
Forskolin-induced swelling in T465N/Q39X rectal organoids following treatment with CFTR modulators. **A** Bright-field microscopy images indicate swelled T465N/Q39X organoids treated as indicated in response to forskolin induction at 0.8 µM with and without 3 µM IVA (VX-770). **B** Quantification of FIS rates. Data are mean ± SD. Kruskal–Wallis test, *p = 0.025, **p = 0.004, ***p = 0.0001 and ****p < 0.0001, modulators-treated organoids versus vehicle (DMSO). **C **Correlation between FIS assay (2 h, 0.8 µM FSK) and Isc values (2D, Ussing) of untreated (vehicle) organoids or organoids cultures pre-stimulated with CFTR modulators: L, T and E/T alone and together with I. The average values of response to therapies measured from rectal organoids swelling at 0.8 µM forskolin were matched with average values of short-circuit measurements (vehicle, I, L, LI, T and ET and ETI-treated organoids). R, P-Pearson correlation coefficient and associated *p*-value; r_s_, p-Spearman’s rank correlation coefficient and associated p-value. Both analyses show a significant correlation between the assays

In this case, organoids had a clear CF phenotype, and we could safely evaluate CFTR functional recovery, if present, by forskolin-induced swelling (FIS) assay [34] to confirm the results obtained with the Ussing chamber technique. The swelling of organoids is an indirect measurement of CFTR activity due to fluid uptake when the CFTR channel is open, as a response to CFTR agonist, forskolin. Hence, the more the organoids swell, the higher the recovery of the mutant CFTR function after CFTR modulator treatment. This assay robustly predicts modulators’ efficacy in vivo [21, 35]. To assess the effect of modulators, T465N/Q39X organoids were pre-incubated for 24 h with L, T and ET and acutely stimulated with forskolin (0.8 µM) alone or in combination with I, using DMSO as vehicle control. The AUC values registered for DMSO-treated organoids was 271 ± 155 in response to 0.8µM of forskolin. As expected, the action of single compounds produced a lower increase of baseline of organoids swelling compared to the effect of double therapy: L/I (AUC 1915 ± 685), T/I (AUC 1591 ± 991). Substantial FIS rates were observed upon exposure to the ETI combination (AUC value 4210 ± 841), confirming the higher efficacy of the triple therapy (Fig. 3A, B). It is noteworthy an excellent correlation between the results of Isc and FIS assays done in colonoids (Fig. 3C) confirming previously reported data [17].

**Fig. 4:**
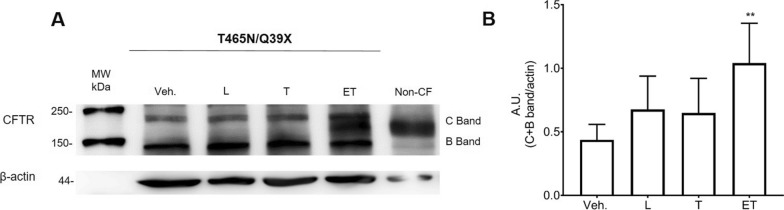
Expression of CFTR protein in T465N/Q39X colonoids. **A** Fully mature CFTR was not detectable at the cell membrane (band C) in vehicle-treated intestinal cells. Treatment for 24 h with 3 µM L and 3 µM T alone does not induce evident amounts of band C that is detectable only with the combination of ET (3 µM each). **B** Densitometric analysis of the western blot data Statistical significance is obtained only for the combined treatment (band B + C, **p = 0.0021, Kruskal–Wallis test). Data are mean ± SD. β-actin polyclonal antibody was used as a loading control. The results of the densitometric analysis shown include the β-actin-normalized data from a minimum of three independent experiments

As the recorded response might be due to either increased activity or increased expression of the missense variant, we investigated the protein expression of T465N/Q39X-CFTR genotype on patient-derived 3D rectal organoids under basal condition and following correctors treatment. We observed that T465N/Q39X lacks the mature, fully glycosylated CFTR protein (C band), in vehicle-treated rectal organoids and that the combined ELEXA/TEZA treatment substantially increased the expression of C band when compared to the rescue ability of single-molecule (Fig. 4). The increase in CFTR band C after ELEXA/TEZA treatment can be attributed to the T465N variant, as class I variants are not the targets of the CFTR modulators used.

## Discussion

CFTR modulator therapies substantially improved the clinical condition of several CF patients, and using an ex vivo organoid-based system has allowed the identification of additional variants as responders to CFTR-targeted treatments. In this study, we assessed CFTR function in rectal organoids derived from three patients carrying the missense variants S737F or T465N; found in heterozygosity with class I variants. We also evaluated the response to clinically approved CFTR modulators and their combination. The presence of nonsense variants in the second alleles known to be not amenable to the correctors/potentiator combination used here allows the isolation of the response of the variants of interest.

S737F was previously characterized in response to CFTR modulators in cultured nasal epithelial cells collected from five subjects, homozygous or heterozygous compound for this variant, with a variable diagnosis for CF disease, all subjects presenting pancreatic sufficiency [29]. Here, we analyzed two of these subjects for which intestinal organoids were available, and we demonstrated that a trend to respond to ETI treatment was present but appeared prominent only in one of the subjects presenting the lowest residual function, while it was reported as significant in nasal cells. The same authors showed that the effect of modulators in recovering S737F-CFTR function was weak compared to the rescue obtained in F508del-CFTR as assessed in the CFBE41o- heterologous expression system. Furthermore, the evaluation of nasal cells derived from a S737F homozygous subject displayed a total CFTR-mediated current comparable to that observed in epithelia from healthy subjects, without any significant improvements following treatment with CFTR modulators. Our electrophysiological measurements in colonoids showed that the S737F/W1282X and S737F/Dele22-24 organoids have a good residual function and displayed an individual variability in CFTR-mediated anion transport. Indeed, while the former case has a residual function indistinguishable from non-CF, the latter has a residual function that was significantly lower, even if within a range considered sufficient to ensure a proper function of the channel (approximately 25% of the non-CF reference). ETI induced a modest increase in CFTR function, which resulted in more evidence for the case carrying the S737F/Dele22-24 genotype.

Visual inspection of the in vitro culture of these organoids revealed a large, visible, internal lumens overlapping to those seen in healthy controls. It is known that the steady-state lumen areas (SLA) is mostly CFTR-driven [20]. Drug-rescued organoids or organoids with residual CFTR function with a swollen phenotype cannot be assessed by FIS because the quantification of organoids swelling is underestimated due to a ceiling effect [15]. Based on that, we performed only the Ussing chamber as a functional assay for these lines. S737F-CFTR is a variant associated with inconclusive diagnosis or with mild CF phenotype and presents residual function of CFTR protein [28]. Its eligibility to ETI treatment (https://pi.vrtx.com/files/uspi_elexacaftor_tezacaftor_ivacaftor.pdf, accessed on January 9th, 2024), as authorized by Food and Drug Administration (FDA), does not guarantee benefits for in vivo treatment. Several works have documented that CFTR genotype is not the only factor involved in drug responsiveness; the individual's genetic background and multiple environmental factors are important elements that contribute to the large inter-patient differences in response registered to the same treatment [38–40]. For instance, CF people harboring F508del- or N1303K-CFTR variants were reported to have variable responses to modulators in vivo and in vitro, demonstrating the importance of using the predictive power of patient-derived rectal organoids for applying the personalized medicine approach in order to optimize the treatment in CF patients. Indeed, functional analysis of CFTR done in rectal organoids in response to drugs has demonstrated a good correlation with relevant clinical parameters [41–43].

These results indicate that substituting serine with phenylalanine does not significantly affect CFTR protein folding and expression at the plasma membrane. Indeed, ETI treatment only marginally promoted the expression of both CFTR forms (B and C bands) of S737F-CFTR as measured in cell line models [29]. Interestingly, our analysis did not detect a significant CFTR dysfunction for S737F/W1282X as seen in nasal epithelial cells derived from the same subject [29]. A quantitative discrepancy in the response between nasal and intestinal cells, with the latter displaying higher Isc values compared with nasal cells, has already been described and has the advantage of increasing the signal-to-noise ratio. It is known, in fact, that rectal organoids present higher CFTR mRNA/protein expression and function compared to human nasal epithelial cells [27]. Nevertheless, considering the whole set of data from nasal and intestinal cells as well as the clinical data, we can conclude that the S737F variant presents a mild effect on CFTR function that is marginally increased in the presence of CFTR modulators in selected cases that need to be identified to optimize the indication to an eventual treatment. Altogether, these data reinforce the reliability of both cell models for diagnostic and theratyping applications and confirm that CFTR-associated currents are highly represented in colonoids.

The second variant studied here was the very rare T465N missense mutation that is not eligible for modulator-based therapies. Furthermore, there is little information about the molecular characterization of this variant and its response to modulators. Interestingly, the biochemical analysis provided evidence that the T465N/Q39X genotype lacks the expression of fully glycosylated CFTR protein at the plasma membrane, suggesting that the amino acid substitution (threonine to asparagine) interferes with CFTR protein folding and trafficking, following the features of a Class II defect for this variant. The functional evaluation of CFTR activity revealed a negligible CFTR function, in line with the lack of mature CFTR protein.

Ivacaftor (VX-770) alone could not potentiate the T465N/Q39X-CFTR function as registered in both assays. The use of single correctors such as lumacaftor (VX-809) and tezacaftor (VX-661) also contributed minimally to the channel restoration with no difference in organoid swelling rates or anion secretion. These correctors alone only barely increased the expression of mature T465N-CFTR protein. We started to detect an increase in T465N-CFTR function with the combined use of a corrector and potentiator. These findings suggest that T465N may also cause defective conductance or reduction of the channel's open probability, typical of a Class III defect. ETI triple therapy increased CFTR-T465N processing and trafficking significantly, enhancing anion transport and FIS rates in vitro. Elexacaftor (VX-445) provided additional positive effects in stabilizing protein maturation and function. Past works have demonstrated the synergistic action of these two correctors through their binding at different sites of the CFTR protein. Elexacaftor (type I corrector) interacts between NBD1 and the transmembrane domain one (TMD1), whereas Tezacaftor (type III corrector) further stabiles NBD1 and improves protein folding by interacting with TM helices 11, 2, and 10 and the lasso motif [44–46]. The highest rescue capacity of ET was previously demonstrated for other class II CF-causing variants [17, 47–49]. To our knowledge, this is the first evidence of the responsiveness of T465N to clinically approved CFTR modulators.

We have shown a significant correlation between 2D monolayer intestinal currents and 3D organoid swelling rates. Our data reinforce the evidence that 2D intestinal short-circuit currents have the same predictive potential to identify clinical responders as seeing for 3D rectal organoids. The advantage of the Ussing technique is the possibility to quantify and monitor the CFTR activity for CFTR variants with substantial residual function, often associated with intestinal organoids with a swollen phenotype, thus overcoming this limitation of the FIS assay.

While several *CFTR* variants different from F508del are included in the U.S. Food and Drug Administration (FDA) list of those approved for CF treatment, in most European countries a variable percentage of people with CF do not have access to ETI because they do not carry the F508del allele on the *CFTR* gene. This is an even more relevant ethical problem in a country like Italy where the percentage of patients with F508del is less than 70% and rare uncharacterized variants are more often identified after gene sequencing. Furthermore, different access to CFTR modulators will widen disparities in CF outcomes between different continents [50]. Ex vivo model can predict in vivo efficacy of CFTR modulator therapy given that drug response correlates with changes in vivo therapeutic endpoints [51–54].

## Conclusion

In conclusion, we demonstrated that S737F/W1282X and S737F/Dele22-24 rectal organoids retain good residual CFTR function that is weakly enhanced by treatment with ETI combination, more evident for the latter case. As such, we might predict a limited clinical benefit of ETI treatment that might become significant in cases with a lower residual function, such as the one with the S737F/Dele22-24 genotype. Clinical treatment results with CFTR modulators in these patients might provide scientific evidence of the superior predictive capability of one of these models (if any) and the importance of a personalized, predictive approach to better guide the clinician to the best possible treatment. Conversely, the theratyping of the very rare T465N variant present in trans with a null allele in patient-derived organoids predicted potential clinical benefits of ETI treatment for patients with this variant. Finally, we confirmed a strong correlation between two functional assays, FIS and Ussing chamber, widely used for drug predictability in vivo, confirming that both methods could be safely used to carry out precision modulator selection useful to drive clinical applications using colonoids.

## Data Availability

The datasets used in and/or analyzed in the current study are available from the corresponding author upon reasonable request. Data sharing is not applicable to this article as no datasets were generated or analyzed during the current study.

## Abbreviations

CF: Cystic fibrosis
CFTR: Cystic fibrosis transmembrane conductance regulator
FDA: Food and Drug Administration (USA)
EMA: European Medical Authorities
CAVD: Congenital absence of the vas deferens
hNEC: Human nasal epithelial cells
NBD1: Nucleotide binding domain
CAVD: Congenital absence of the vas deferens
ETI: Elexacaftor/Tezacaftor/Ivacaftor
AUC: Area under the curve
CRMS: CFTR-related metabolic syndrome
CFSPID: CF screen positive, inconclusive diagnosis
SC: Sweat chloride
IRT: Immunoreactive trypsinogen
NBS: Newborn bloodspot screening
BMI: Body mass index
FEV_1_: Forced expiratory volume in the 1st second
MSSA: Methicillin-susceptible Staphylococcus aureus
PS: Pancreatic sufficiency
PI: Pancreatic insufficiency
